# Glycoproteomic characterization of carriers of the CD15/Lewis^x ^epitope on Hodgkin's Reed-Sternberg cells

**DOI:** 10.1186/1471-2091-12-13

**Published:** 2011-03-24

**Authors:** Alex S Powlesland, Maria Marcela Barrio, José Mordoh, Paul G Hitchen, Anne Dell, Kurt Drickamer, Maureen E Taylor

**Affiliations:** 1 Division of Molecular Biosciences, Department of Life Sciences, Imperial College, London SW7 2AZ, UK; 2 Centro de Investigaciones Oncológicas FUCA, Zabala 2836, 1426 Buenos Aires, Argentina; 3 Fundación Instituto Leloir-IIBBA CONICET, Patricias Argentinas 435, 1405 Buenos Aires, Argentina; 4 Centre for Integrative Systems Biology, Department of Life Sciences, Imperial College, London SW7 2AZ, UK

## Abstract

**Background:**

The Lewis^x ^trisaccharide, also referred to as the CD15 antigen, is a diagnostic marker used to distinguish Hodgkin's lymphoma from other lymphocytic cancers. However, the role of such fucosylated structures remains poorly understood, in part because carriers of Lewis^x ^structures on Hodgkin's Reed-Sternberg cells have not been identified.

**Methods:**

GalMBP, an engineered carbohydrate-recognition protein that binds selectively to oligosaccharides with paired terminal galactose and fucose residues, has been used in conjunction with proteomic and glycomic analysis to identify glycoprotein carriers of Lewis^x ^and related glycan structures in multiple Hodgkin's Reed-Sternberg cell lines.

**Results:**

Multiple glycoproteins that bind to GalMBP and carry CD15/Lewis^x ^have been identified in a panel of six Reed-Sternberg cell lines. The most commonly identified Lewis^x^-bearing glycoproteins are CD98hc, which was found in all six cell lines tested, and intercellular adhesion molecule-1 and DEC-205, which were detected in five and four of the lines, respectively. Thus, several of the most prominent cell adhesion molecules on the lymphomas carry this characteristic glycan epitope. In addition, the Hodgkin's Reed-Sternberg cell lines can be grouped into subsets based on the presence or absence of less common Lewis^x^-bearing glycoproteins.

**Conclusions:**

CD98 and intercellular adhesion molecule-1 are major carriers of CD15/Lewis^x ^on Reed-Sternberg cells. Binding of DC-SIGN and other glycan-specific receptors to the Lewis^x ^epitopes on CD98 and intercellular adhesion molecule-1 may facilitate interaction of the lymphoma cells with lymphocytes and myeloid cells in lymph nodes.

## Background

The Lewis^x ^blood group epitope, also referred to as the CD15 antigen, has been reported on many different cancers and cancer cell lines including Hodgkins lymphomas, a common form of lymphocytic cancer. The presence of Lewis^x ^has been used as a marker for the neoplastic tumour cells of Hodgkins lymphoma, referred to as Hodgkins Reed-Sternberg (HRS) cells. HRS cells form a relatively small population of the tumour mass, with the remaining cells consisting of non-neoplastic reactive cells including T lymphocytes, granulocytes, macrophages and plasma cells [1,2]. Crosslinking of HRS cell-surface molecules containing Lewis^x^, using anti-Lewis^x ^antibodies, stimulates cellular signaling through the tyrosine phosphorylation of proteins including c-Cbl [3], suggesting that identification of protein carriers of Lewis^x ^on HRS cells may provide insight into how cellular activation is achieved.

The C-type (Ca^2+^-dependent) carbohydrate-recognition domain of serum mannose-binding protein, which normally binds to mannose-containing oligosaccharides characteristic of pathogens, can be re-engineered to bind galactose-containing glycans [4,5]. Glycan array analysis reveals that the modified protein, referred to as galactose-specific mannose-binding protien (GalMBP), binds preferentially to oligosaccharides in which terminal galactose residues are adjacent to terminal fucose residues, as in the Lewis^x ^blood group epitope [6]. The specificity of GalMBP indicated that it would be a useful tool for probing the way that Lewis^x ^is presented on the surface of Reed-Sternberg cells.

By combining affinity purification on immobilized GalMBP with glycomics and proteomics, several cell surface molecules on HRS cells have been found to bear the Lewis^x ^epitope, with the heavy chain of CD98 being a common carrier on multiple HRS cell lines.

## Methods

### Cell culture

HRS cell lines L-428, KMH-2, L-1236, L-540, HDLM-2 and U-HO1 were purchased from the DSMZ (Deutsche Sammlung von Mikroorganismen und Zellkulturen GmbH), Braunschweig, Germany, which provided characterization using antibody reactivity of cell surface markers, PCR of minisatellite markers, isoelectric focusing of malate dehydrogenase and aspartate aminotransferase, and cytogenetics. Cell lines L-428, KM-H2 and L-1236 were grown in RPMI-1640 medium supplemented with 10% fetal calf serum, 2 mM glutamine, 100 U/ml penicillin, and 100 μg/ml streptomycin. Cell lines L-540 and HDLM-2 were grown in the same medium but with 20% fetal calf serum. Cell line U-HO1 was grown in 1:4 Iscove's modified Dulbecco's medium:RPMI-1640 medium supplemented with 20% fetal calf serum, 2 mM glutamine, 100 U/ml penicillin, and 100 μg/ml streptomycin.

### Purification of membrane glycoproteins on immobilized GalMBP

Cells grown to 0.5 - 1 × 10^6 ^cells/ml in 100 ml of medium were harvested by centrifugation at 450 × g for 2 min, washed twice in 10 ml of phosphate-buffered saline, resuspended in 10 ml of cell lysis buffer (150 mM NaCl; 25 mM Tris-Cl, pH 7.8; 2 mM CaCl_2_; 1% Triton X-100) and protease inhibitors (Cocktail mix 1, Merck, Nottingham, UK), sonicated for 10 s and incubated on ice for 30 min. Lysate was precleared by centrifugation at 100,000 × g for 15 min at 4°C and passed over 2-ml GalMBP-agarose columns [6] that were washed with 5 ml of loading buffer (150 mM NaCl; 25 mM Tris-Cl, pH 7.8; 2 mM CaCl_2_; 0.1% Triton X-100) and eluted with 1-ml fractions of elution buffer (150 mM NaCl; 25 mM Tris-Cl, pH 7.8; 2.5 mM EDTA; 0.1% Triton X-100). Pooled elution fractions were brought to 25 mM CaCl_2 _and passed over a second GalMBP column that was washed with 5 ml of high salt buffer (1.25 M NaCl; 25 mM Tris-Cl, pH 7.8; 25 mM CaCl_2_; 0.1% Triton X-100) and 5 ml of loading buffer followed by elution as above.

### Gel analysis of GalMBP ligands

Samples were analyzed on 15% SDS-polyacrylamide gels that were electroblotted onto Protran nitrocellulose membranes (Whatman Plc, Kent, UK) [7]. For probing with ^125^I-GalMBP [8], membranes were blocked overnight at 4°C with 2% hemoglobin in binding buffer (150 mM NaCl; 25 mM Tris-Cl, pH 7.8; 2 mM CaCl_2_) and incubated with ^125^I-GalMBP for 2 h at room temperature with gentle shaking in the same buffer. Following four washes with binding buffer, radioactivity on membranes was detected using a Phosphorimager SI from Molecular Dynamics (Sunnyvale, USA). Probing with anti-Lewis^x ^antibodies MCS-1 (Quest Biomedicals Ltd, West Midlands, UK) and FC-2.15 was performed as previously described [9].

### Glycan analysis

N-linked glycans released from tryptic peptides by digestion with peptide:N-glycosidase F were purified using a Sep-Pak C18 reverse phase cartridge and permethylated using the anhydrous sodium hydroxide method [10]. Following a further round of Sep-Pak purification, permethylated glycans were dissolved in 10 μl of methanol:H_2_O (4:1), mixed with an equal volume of 2,5-dihydroxybenzoic acid matrix and subjected to matrix-assisted laser desorption ionization mass spectrometry (MALDI MS) profiling with further tandem mass spectrometry (MS/MS) analysis of selected ions [11].

### Proteomic analysis

Proteins resolved on SDS-polyacrylamide gels were excised, and subjected to proteomic analysis by in-gel trypsin digestion [6] using MALDI MS profiling complemented with MS/MS sequencing of the ten most abundant ions in each sample on an Applied BioSystems 4800 MALDI tandem time-of-flight mass spectrometer. For in-solution trypsin digestion, fractions were precipitated with trichloroacetic acid, dissolved in 8 M urea containing 10 mM HEPES, pH 8.0, reduced, alkylated, and digested in solution with trypsin [12]. Tryptic peptides were dried, desalted, and purified by nano-liquid chromatography using a 15 μm × 15 cm Pepmap analytical C18 nanocapillary column on an Ultimate 3000 liquid chromatography system (LC Packings, Dionex, Sunnyvale, USA) eluted with a gradient from 8-45% acetonitrile over 60 min at a flow rate of 0.3 μl/min [6]. Eluted peptides were detected by MALDI MS profiling and sequenced by MS/MS.

## Results

### Affinity purification of glycoproteins that bear Lewis^x ^structures on HRS cells

**Figure 1:**
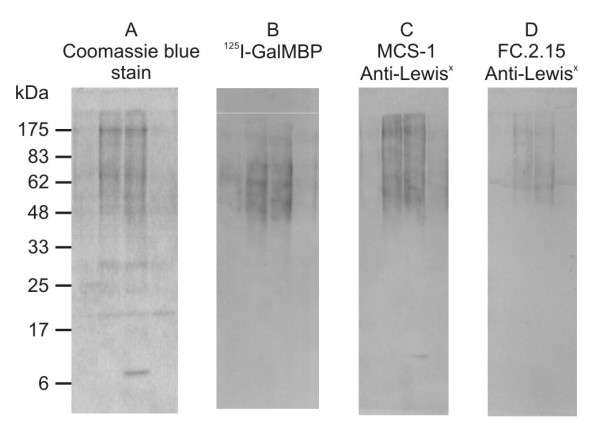
**Analysis of GalMBP ligands from L-428 cells with anti-Lewis^x ^antibodies**. Elution fractions from solubilized L-428 cells purified over a column of immobilized GalMBP were analyzed on 15% SDS-polyacrylamide gels followed by Coomassie blue staining or electroblotting onto nitrocellulose and probing with anti-Lewis^x ^antibodies and radiolabelled GalMBP. The different relative intensities of some of the bands suggest that GalMBP and the antibodies bind differentially to glycans.

L-428 cells, which are model HRS cells on which Lewis^x ^expression has been documented using monoclonal antibodies [3], were used to test the utility of GalMBP for purification of glycoproteins that carry Lewis^x^. Glycoproteins in detergent extracts of L-428 cell that bound to immobilized GalMBP in the presence of Ca^2+ ^were eluted with EDTA and analyzed by SDS-polyacrylamide gel electrophoresis, demonstrating the presence of multiple potential ligands covering a broad distribution of molecular weights ranging from 48 kDa to well over 175 kDa (Figure 1A). Probing of western blots confirmed that these eluted glycoproteins bind to radiolabelled GalMBP (Figure 1B) and the presence of glycans containing Lewis^x ^was also demonstrated by probing the blots with two anti-Lewis^x ^antibodies (Figure 1C,D).

**Figure 2:**
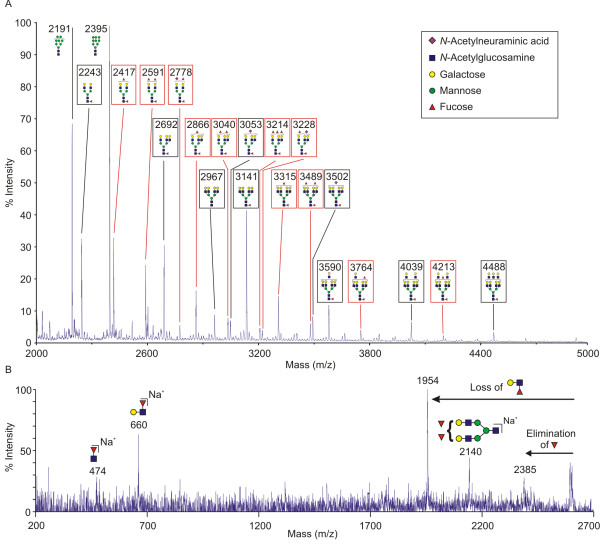
**Mass spectrometric analysis of N-linked glycans from GalMBP ligands on L-428 cells**. Purified GalMBP ligands were digested with trypsin and N-linked glycans were released by peptide:N-glycosidase F. N-linked glycans were permethylated and subjected to Sep-Pak purification. A. The MALDI MS spectrum of one of the elution fractions from the Sep-Pak is shown. All labeled ions were subjected to MS/MS analysis. The schemes for each ion represent the most likely structure based on the fit between the calculated composition and the m/z (z = 1) ratio of the ions detected, taking into account the biosynthetic pathways of N-glycosylation in addition to the MS and MS/MS data collected. Structures in red boxes contain terminal fucose residues. B. An example of the MALDI MS/MS spectra, showing fragmentation of the 2591 molecular ion. The presence of major fragment ions at 1954 and 660 confirms the presence of terminal Lewis^x ^or Lewis^a ^structures. Specific evidence for the presence of Lewis^x ^structures is provided by elimination of fucose from the 3 position of GlcNAc to form the 2385 fragment ion. The presence of unfucosylated glycans that are suboptimal ligands for GalMBP, as well as high-mannose oligosaccharides that do not bind GalMBP at all, is presumed to reflect the presence of heavily glycosylated glycoproteins that also contain complex-type glycans bound by GalMBP as previously observed for breast cancer cells [6].

MALDI MS analysis of N-glycans released from the pool of glycoproteins eluted from the GalMBP column revealed the presence of glycans containing up to 3 fucose residues (Figure 2A) and MS/MS analysis of the major molecular ions led to identification of bi-, tri- and tetra-antennary complex glycans that are terminally fucosylated (Figure 2B). These glycans gave fragment ions at *m/z *660, corresponding to terminal FucHexHexNAc, which would represent terminal Lewis^a ^or Lewis^x ^trisaccharides (Figure 2B). The MS/MS fragmentation also provided evidence that a portion of these termini are in the form Lewis^x ^epitopes, consistent with the antibody binding data. Comparing related glycan structures indicates that a substantial proportion of each class of branched structure contains terminal fucosylated structures. For example, the relative intensities of the molecular ions at *m/z *2416 and 2591, which correspond to bi-antennary glycans containing two and three fucose residues, is comparable to that of the molecular ion *m/z *2243, corresponding to a bi-antennary glycan without terminal fucosylation. Larger glycans present include tetra-antennary structures that contain one or two fucosylated branches and glycans with poly-*N*-acetyllactosamine extensions, many of which also bear terminal fucose residues.

### Proteomic identification of GalMBP ligands on L-428 cells

**Figure 3:**
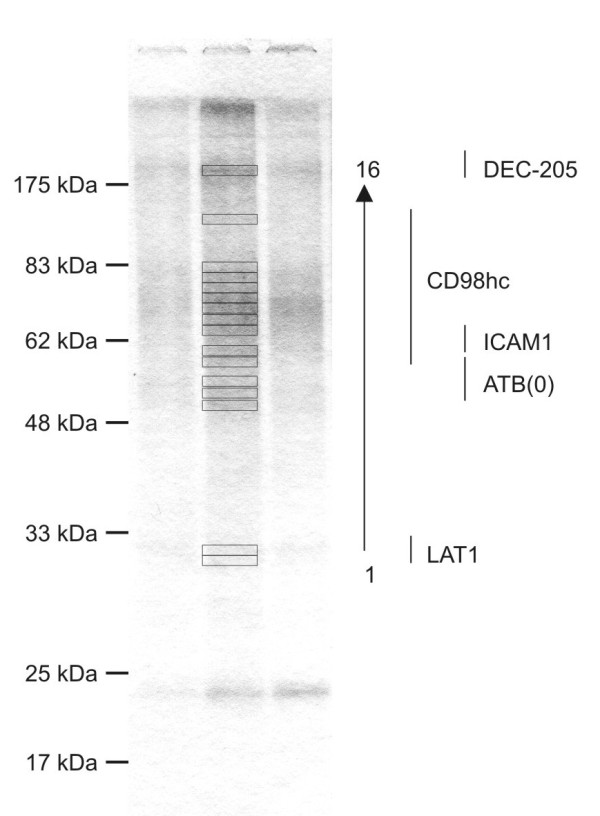
**GalMBP ligands from L-428 cells. Cell extracts were fractionated on columns of immobilized GalMBP**. Proteins present in elution fractions from the GalMBP column were resolved on 15% SDS-polyacrylamide gels, excised, and subjected to proteomic analysis by in-gel trypsin digestion. Proteins present in bands 1-16, identified by mass spectrometry following trypsin digestion, are listed in the right hand column and in Additional file 1.

The identity of glycoproteins that bear this spectrum of Lewis^x^-containing glycans was initially investigated by proteomic analysis of proteins fractioned by SDS-polyacrylamide gel electrophoresis. Despite the presence of overlapping bands, probably due to heterogeneity of glycans present on individual glycoproteins, multiple glycoproteins were identified, including the dendritic and epithelial cell receptor DEC-205 (CD205) and intercellular adhesion molecule 1 (ICAM-1) (Figure 3 and Additional file 1). DEC-205 and ICAM-1 are both heavily glycosylated proteins known to be expressed in HRS cells [13,14]. The heavy chain of the cell surface molecule CD98 (CD98hc) was identified across a region of the gel between approximately 75 and 100 kDa and the accompanying small chain LAT1 was also detected, but below confidence levels for the analysis (Additional file 1). Both LAT1 and the ATB(0) protein, which was detected just above the confidence threshold, are amino acid transporters. The average distance between glycosylated asparagine residues in CD98hc is approximately 26 Å, so that terminal galactose residues on N-linked glycans attached at these sites could bridge the distance between binding sites in a GalMBP trimer [15], which may contribute to the effectiveness with which CD98hc can be isolated from multiple HRS cell lines using GalMBP.

In an alternative approach to the proteomic analysis, glycoproteins purified on GalMBP were identified directly by trypsin digestion of the pooled elution fractions. The resulting peptides were purified by liquid chromatography and individual peptides were identified by MALDI MS analysis and sequenced by collision-induced dissociation followed by tandem mass spectrometry. In agreement with the in-gel analysis, CD98hc, DEC-205 and ICAM-1 were identified with the highest levels of confidence (Additional file 2), thus validating the batch digestion approach as a means of glycoprotein identification. Because of the increased sensitivity and reduced background of the batch analysis, several additional glycoproteins not detected by the in-gel analysis, including cell-surface receptors CD70, CD86, CD147 and HLA-DR, were also identified in the elution fractions.

### Identification of common GalMBP ligands on HRS cell lines

**Figure 4:**
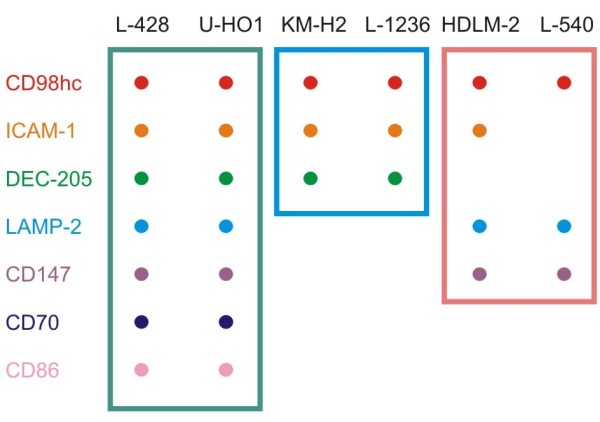
**Glycoproteins found in multiple HRS cell lines**. Proteins present in at least two cell lines in Additional file 2 are indicated by dots. Colored boxes highlight subgroups within the panel of cell lines.

Based on the results of the glycomic and proteomic analysis of the L-428 cells, fractionation on GalMBP followed by batchwise proteomic analysis was used for screening multiple cell lines to characterize the spectrum of glycoprotein carriers of Lewis^x ^in Hodgkin's lymphoma. Analysis of a panel of five further HRS cell lines showed a pattern of common Lewis^x ^carrier glycoproteins found in the majority of the cell lines (Figure 4 and Additional file 2). The most commonly identified glycoproteins were CD98hc, identified in all of the cell lines, ICAM-1, found in all except one line and DEC-205, detected in four of the six lines. These results suggest that these proteins often present Lewis^x ^structures and may indicate a potential role for the glycan structure in the function of these proteins on HRS cells. However, the data also indicated that there is variability between cell lines and that they can be grouped based on the presence of different sets of carrier glycoproteins in the GalMBP elution fractions (Figure 4), although each cell line appears to have some unique characteristics.

## Discussion

Glycoproteins identified in HRS cells by GalMBP affinity chromatography provide potential clues about the role of Lewis^x^-containing glycans in the pathology of Hodgkin's lymphoma. Two of the major ligands identified, CD98hc and ICAM-1, participate in integrin-mediated adhesion [16]. Binding of Lewis^x^-bearing glycans on these molecules by glycan-specific receptors on the surfaces of lymphocytes and myeloid cells may facilitate their binding to counter-receptors on target cells by bringing them into close proximity to their ligands. For example, DC-SIGN binding to Lewis^x^-containing glycans [17], could initiate interactions between HRS cells and dendritic cells. The lysosome-associated membrane proteins LAMP1 and LAMP2 bind to the GalMBP columns. These heavily glycosylated proteins predominantly reside in lysosomes, but have also been implicated in adhesion of activated lymphocytes to endothelial cells [18].

Identification of ICAM-1 and CD98hc as major carriers of Lewis^x ^structures may explain, in part, why antibodies specific to Lewis^x ^structures are able to activate tyrosine kinase signaling pathways in HRS cell lines [3], because both of these receptors can transmit activation signals following binding by extracellular ligands [19,20]. Lewis^x^-containing glycoproteins on HRS cells could act in a manner analogous to the way that Lewis^x ^structures on parasites such as *Schistosoma mansoni *induce a Th2 response by binding to C-type lectins and modulating cytokine production [21]. For example, the presence of Lewis^x^-bearing glycans on glycoproteins that are involved in co-stimulation of CD4^+ ^T cells could facilitate polarization of activated T cells towards a Th2 response [22], thus maintaining an inflammatory microenvironment within the tumor and suppressing potential anti-tumor activity of Th1 cells.

The ability to purify both major and minor populations of Lewis^x^-containing glycoproteins on immobilized GalMBP has revealed differences in the panel of glycoproteins purified from each cell line. These variations define subsets of HRS cell lines and suggest that GalMBP may be used to classify HRS cell lines into subgroups that present Lewis^x ^on specific protein scaffolds.

## Conclusions

Affinity purification on immobilized GalMBP combined with glycomics and proteomics has been validated as a means of characterizing glycoproteins on HRS cells that bear the Lewis^x ^epitope. These glycoproteins, including CD98 and ICAM-1, are potential targets for both anti-CD15 antibodies and for endogenous glycan-binding receptors.

## List of Abbreviations

HRS: Hodgkin's Reed-Sternberg; GalMBP: galactose-binding variant of mannose-binding protein; MALDI: matrix-assisted laser desorption/ionization; MS: mass spectrometry; ICAM: intercellular adhesion molecule.

## Competing interests

The authors declare that they have no competing interests.

## Authors' contributions

ASP carried out the glycoprotein isolation and mass spectrometry analysis, MMB and JM developed and produced the FC-2.15 antibody, PGH and AD participated in the collection and interpretation of mass spectrometry data, ASP, KD, and MET participated in the experimental design, interpretation of the results and preparation of the manuscript. All authors read and approved the final manuscript.

## Supplementary Material

Additional file 1 **MALDI MS analysis of tryptic fragments from L-428 cell proteins**. Bands in Figure 2 were identified by MS and MS/MS analysis of tryptic fragments and searching of the SwissProt protein database. Click here for file

Additional file 2 **GalMBP ligands from HRS cell lines identified by in-solution digestion followed by liquid chromatography and MS/MS analysis**. The SwissProt database was searched for sequences consistent with the MS/MS fragment ions for cell lines L-428, HDLM-2, KM-H2, L-1236, U-H01, and L-540. Click here for file

## References

[B1] Hall PA D'Ardenne AJ Value of CD15 immunostaining in diagnosing Hodgkin's disease: a review of published literature J Clin Pathol 1987 40 1298 304 10.1136/jcp.40.11.1298 3320093 PMC1141228

[B2] Pellegrini W Bresciani G De Zorzi A Marocolo D Ungari M Facchetti F MMA monoclonal antibody is a superior anti-CD15 reagent for the diagnosis of classical Hodgkin's lymphoma? Haematologica 2007 92 708 9 10.3324/haematol.11002 17488702

[B3] Ohana-Malka O Benharroch D Isakov N Prinsloo I Shubinsky G Sacks M Gopas J Selectins and anti-CD15 (Lewis^x/a^) antibodies transmit activation signals in Hodgkin's lymphoma-derived cell lines Exp Hematol 2003 31 1057 65 10.1016/S0301-472X(03)00237-6 14585370

[B4] Drickamer K Engineering galactose-binding activity into a C-type mannose-binding protein Nature 1992 360 183 6 10.1038/360183a0 1279438

[B5] Iobst ST Drickamer K Binding of sugar ligands to Ca^2+^-dependent animal lectins. II. Generation of high-affinity galactose binding by site-directed mutagenesis J Biol Chem 1994 269 15512 9 8195195

[B6] Powlesland AS Hitchen PG Parry S Graham SA Barrio MM Elola MT Mordoh J Dell A Drickamer K Taylor ME Targeted glycoproteomic identification of cancer cell glycosylation Glycobiology 2009 19 899 909 10.1093/glycob/cwp065 19433864 PMC2704901

[B7] Burnette WN "Western blotting": electrophoretic transfer of proteins from sodium dodecyl sulfate--polyacrylamide gels to unmodified nitrocellulose and radiographic detection with antibody and radioiodinated protein A Anal Biochem 1981 112 195 203 10.1016/0003-2697(81)90281-5 6266278

[B8] Coombs PJ Graham SA Drickamer K Taylor ME Selective binding of the scavenger receptor C-type lectin to Lewis^x ^trisaccharide and related glycan ligands J Biol Chem 2005 280 22993 9 10.1074/jbc.M504197200 15845541

[B9] Elola MT Capurro MI Barrio MM Coombs PJ Taylor ME Drickamer K Mordoh J Lewis^x ^antigen mediates adhesion of human breast carcinoma cells to activated endothelium. Possible involvement of the endothelial scavenger receptor C-type lectin Breast Cancer Res Treat 2007 101 161 74 10.1007/s10549-006-9286-9 16850248 PMC2288708

[B10] Sutton-Smith M Dell A Celis, J Analysis of carbohydrates/glycoproteins by mass spectrometry Cell Biology: A Laboratory Handbook 2005 4 3 San Diego: Academic Press 415 25

[B11] Domon B Costello CE A systematic nomenclature for carbohydrate fragmentations in FAB-MS/MS spectra of glycoconjugates Glycoconjugate J 1988 5 397 409 10.1007/BF01049915

[B12] Foster LJ Mann M Celis, J Protein identification and sequencing by mass spectrometry Cell Biology: A Laboratory Handbook 2005 4 3 San Diego: Academic Press 363 9

[B13] Kato M Khan S Gonzalez N O'Neill BP McDonald KJ Cooper BJ Angel NZ Hart DN Hodgkin's lymphoma cell lines express a fusion protein encoded by intergenically spliced mRNA for the multilectin receptor DEC-205 (CD205) and a novel C-type lectin receptor DCL-1 J Biol Chem 2003 278 34035 41 10.1074/jbc.M303112200 12824192

[B14] Uchihara JN Matsuda T Okudaira T Ishikawa C Masuda M Horie R Watanabe T Ohta T Takasu N Mori N Transactivation of the ICAM-1 gene by CD30 in Hodgkin's lymphoma Int J Cancer 2006 118 1098 107 10.1002/ijc.21427 16152613

[B15] Weis WI Drickamer K Trimeric structure of a C-type mannose-binding protein Structure 1994 2 1227 40 10.1016/S0969-2126(94)00124-3 7704532

[B16] Feral CC Nishiya N Fenczik CA Stuhlmann H Slepak M Ginsberg MH CD98hc (SLC3A2) mediates integrin signaling Proc Natl Acad Sci USA 2005 102 355 60 10.1073/pnas.0404852102 15625115 PMC544283

[B17] Guo Y Feinberg H Conroy E Mitchell DA Alvarez R Blixt O Taylor ME Weis WI Drickamer K Structural basis for distinct ligand-binding and targeting properties of the receptors DC-SIGN and DC-SIGNR Nat Struct Mol Biol 2004 11 591 8 10.1038/nsmb784 15195147

[B18] Kannan K Stewart RM Bounds W Carlsson SR Fukuda M Betzing KW Holcombe RF Lysosome-associated membrane proteins h-LAMP1 (CD107a) and h-LAMP2 (CD107b) are activation-dependent cell surface glycoproteins in human peripheral blood mononuclear cells which mediate cell adhesion to vascular endothelium Cell Immunol 1996 171 10 9 10.1006/cimm.1996.0167 8660832

[B19] Lawson C Wolf S ICAM-1 signaling in endothelial cells Pharm Rep 2009 61 22 32 10.1016/s1734-1140(09)70004-0 19307690

[B20] Rintoul RC Buttery RC Mackinnon AC Wong WS Mosher D Haslett C Sethi T Cross-linking CD98 promotes integrin-like signaling and anchorage-independent growth Mol Biol Cell 2002 13 2841 52 10.1091/mbc.01-11-0530 12181350 PMC117946

[B21] Geijtenbeek TBH Gringhuis SI Signalling through C-type lectin receptors: shaping immune responses Nat Rev Immunol 2009 9 465 79 10.1038/nri2569 19521399 PMC7097056

[B22] Kuppers R The biology of Hodgkin's lymphoma Nat Rev Cancer 2009 9 15 27 10.1038/nrc2542 19078975

