# Supplementary material for: Glycoproteomic characterization of carriers of the CD15/Lewisx epitope on Hodgkin's Reed-Sternberg cells
Source: BMC Biochem. 2011 Mar 24;12:13. doi: 10.1186/1471-2091-12-13 (PMC3071785; doi:10.1186/1471-2091-12-13)
Supplement: Additional file 1 — MALDI MS analysis of tryptic fragments from L-428 cell proteins. Bands in Figure 2 were identified by MS and MS/MS analysis of tryptic fragments and searching of the SwissProt protein database. [file 1471-2091-12-13-S1.PDF]

Additional file 1. MALDI MS analysis of tryptic fragments from L-428 cell proteins

Analysis was conducted with GPS Explorer software version 3.6 (Applied Biosystems, Foster City, USA). For peptide mass fingerprint data, peak picking was conducted with a signal-to-noise threshold of 10, with no contaminant ions excluded, using a peak density filter of 50 peaks per 200 Da, and a maximum of 65 peaks were considered. For MS/MS experiments, peak list generation and database searching were conducted with the default parameters. Both MS and MS/MS data were used to search the *Homo sapiens* portion of the SwissProt protein database using Mascot software (www.matrixscience.com) with the following parameters: monoisotopic peptide masses, allowing for partial oxidation of methionine residues and carboxymethylation of cysteine residues, mass tolerance of 75 ppm. and fragment ion tolerance of 0.1 Da. Tryptic digests were assumed to have no missed cleavages. Protein identifications with a Mowse protein score, equal to -10 x Log(*P*), where *P* is the probability that the observed match is a random event, of greater than 55 ($p < 0.05$) were considered significant. Band numbers refer to Figure 2.

Band No.	SwissProt accession number	Protein name	Precursor mass	Peptide score	Peptides matched	Sequence Coverage (%)
1	Q8WWH5	Probable tRNA pseudouridine synthase 1	37253	65	9	32
2	Q01650	Large neutral amino acids transporter small subunit 1 (CD98lc)	54974	54	6	17
3	P07437	Tubulin beta chain	49671	83	10	29
	Q9UPN3	Microtubule-actin crosslinking factor 1	620418	78	40	10
4	P68363	Tubulin alpha chain	50152	99	12	41
5	P68363	Tubulin alpha chain	50152	95	10	30
	P04264	Keratin, type II cytoskeletal 1	66018	90	18	29
6	P04264	Keratin, type II cytoskeletal 1	66018	57	12	25
7	P04264	Keratin, type II cytoskeletal 1	66018	82	15	29
8	Q15758	Neutral amino acid transporter B(0)	56598	58	9	22
9	Q15758	Neutral amino acid transporter B(0)	56598	58	9	18
10	P08195	4F2 cell-surface antigen heavy chain (CD98hc)	57909	77	14	27
	Q15758	Neutral amino acid transporter B(0)	56598	58	9	18
11	P08195	4F2 cell-surface antigen heavy chain (CD98hc)	57909	110	13	27
	P05362	Intercellular-adhesion molecule 1 (ICAM-1)	57825	89	10	20
	Q96PK2	Microtubule-actin crosslinking factor 4	670151	63	38	9
12	P08195	4F2 cell-surface antigen heavy chain (CD98hc)	57909	83	14	28
13	P08195	4F2 cell-surface antigen heavy chain (CD98hc)	57909	111	14	30
	Q9UPN3	Microtubule-actin crosslinking factor 1	620418	70	36	9
14	P08195	4F2 cell-surface antigen heavy chain (CD98hc)	57909	99	16	35
15	P08195	4F2 cell-surface antigen heavy chain (CD98hc)	57909	60	12	24
16	O60449	Lymphocyte antigen 75 (DEC-205)	198271	81	20	13
