# Supplementary material for: Glycoproteomic characterization of carriers of the CD15/Lewisx epitope on Hodgkin's Reed-Sternberg cells
Source: BMC Biochem. 2011 Mar 24;12:13. doi: 10.1186/1471-2091-12-13 (PMC3071785; doi:10.1186/1471-2091-12-13)
Supplement: Additional file 2 — GalMBP ligands from HRS cell lines identified by in-solution digestion followed by liquid chromatography and MS/MS analysis. The SwissProt database was searched for sequences consistent with the MS/MS fragment ions for cell lines L-428, HDLM-2, KM-H2, L-1236, U-H01, and L-540. [file 1471-2091-12-13-S2.PDF]

Additional file 2. GalMBP ligands from HRS cell lines identified by in-solution digestion followed by liquid chromatography and MS/MS analysis

The SwissProt database was searched for sequences consistent with the MS/MS fragment ions. The parameters for database searching were modified compared to those in Table 1 to increase the fragment ion tolerance to 0.5. Proteins listed contain three or more peptides with ion scores higher than 29, which indicates identity or extensive sequence similarity ($p<0.05$).

L-428 cells

Protein Accession Number (Swissprot)	Protein Name	Peptides matched	Total Ion Score
4F2_HUMAN	CD98 heavy chain	16	1104
LY75_HUMAN	DEC-205 (CD205)	12	568
ICAM1_HUMAN	Intercellular adhesion molecule 1; ICAM-1 (CD54)	7	389
TBA1B_HUMAN	Tubulin alpha chain	6	317
TBA1C_HUMAN	Tubulin alpha chain	6	303
CD70_HUMAN	CD70	5	298
2DRA_HUMAN	HLA class II DR alpha chain	5	235
S4A7_HUMAN	Sodium bicarbonate cotransporter 3	5	230
TBB5_HUMAN	Tubulin beta chain	4	209
BASI_HUMAN	Basigin (CD147)	3	241
LAMP2_HUMAN	Lysosome-associated membrane protein-2 (LAMP2)	3	171
2B32_HUMAN	HLA class II DRB3-2 beta chain	3	140
CD86_HUMAN	CD86 (B7.2)	3	127

HDLM-2 Cells

Protein Accession Number (Swissprot)	Protein Name	Peptides matched	Total Ion Score
4F2_HUMAN	CD98 heavy chain	8	429
K2C1_HUMAN	Keratin, type II cytoskeletal 1	5	281
BASI_HUMAN	Basigin (CD147)	4	188
CALX_HUMAN	Calnexin	4	164
AAAT_HUMAN	Neutral amino acid transporter B(0)	3	161
SCRB2_HUMAN	Lysosome membrane protein 2	3	153
LAMP2_HUMAN	Lysosome-associated membrane protein-2 (LAMP2)	3	138
K1C10_HUMAN	Keratin, type I cytoskeletal 10	3	113
ICAM1_HUMAN	ICAM-1 (CD54)	3	111

KM-H2 Cells

Protein Accession Number (Swissprot)	Protein Name	Peptides matched	Total Ion Score
ICAM1_HUMAN	ICAM-1 (CD54)	8	616
4F2_HUMAN	CD98 heavy chain	7	444
2B1B_HUMAN	HLA class II DRB1 beta chain	6	434
2DRA_HUMAN	HLA class II DR alpha chain	6	376
LY75_HUMAN	DEC-205 (CD205)	6	361
CD45_HUMAN	Leukocyte common antigen CD45	6	248
GRP78_HUMAN	78 KDa glucose-regulated protein	5	244
CD70_HUMAN	CD70	4	231
K2C1_HUMAN	Keratin, type II cytoskeletal 1	4	219
1B35_HUMAN	HLA Class I, B	3	207
1C12_HUMAN	HLA Class I, C	3	186
CLC4M_HUMAN	DC-SIGNR (CD299)	3	165
PRG4_HUMAN	Proteoglycan 4	3	160

L-1236 Cells

Protein Accession Number (Swissprot)	Protein Name	Peptides matched	Total Ion Score
PECA1_HUMAN	Platelet endothelial cell adhesion molecule PECAM-1 (CD31)	8	601
ICAM1_HUMAN	ICAM-1 (CD54)	7	454
4F2_HUMAN	CD98 heavy chain	7	436
DPP4_HUMAN	Dipeptidyl peptidase 4	7	388
CD45_HUMAN	Leukocyte common antigen (CD45)	6	342
K1C10_HUMAN	Keratin, type I cytoskeletal 10	6	329
K2C1_HUMAN	Keratin, type II cytoskeletal 1	5	397
LY75_HUMAN	DEC-205 (CD205)	5	224
TBA1A_HUMAN<br>TBA1B_HUMAN<br>TBA1C_HUMAN	Tubulin alpha-1A chain<br>Tubulin alpha-1B chain<br>Tubulin alpha-1C chain	4	281
AAAT_HUMAN	Neutral amino acid transporter B(0)	4	241
ADA_HUMAN	Adenosine deaminase	4	224
1A02_HUMAN	HLA class I, A	4	180
TBB5_HUMAN	Tubulin beta chain	3	167
SLAF1_HUMAN	Signalling lymphocytic activation molecule (CD150)	3	136

U-H01 Cells

Protein Accession Number (Swissprot)	Protein Name	Peptides matched	Total Ion Score
LY75_HUMAN	DEC-205 (CD205)	30	1869
4F2_HUMAN	CD98 heavy chain	12	818
LCAP_HUMAN	Leucyl-cystinyl aminopeptidase	10	641
ICAM1_HUMAN	ICAM-1 (CD54)	10	535
PPBT_HUMAN	Alkaline phosphatase, tissue non-specific isozyme	8	432
LAMP1_HUMAN	Lysosome-associated membrane protein-1 (LAMP1)	6	405
CD70_HUMAN	CD70	6	357
TBA1A_HUMAN<br>TBA1B_HUMAN<br>TBA1C_HUMAN	Tubulin alpha-1A chain<br>Tubulin alpha-1B chain<br>Tubulin alpha-1C chain	4	278
ATA1_HUMAN	Na/K-transporting ATPase subunit alpha-1	4	181
MRP1_HUMAN	Multidrug resistance-associated protein 1	4	161
LAMP2_HUMAN	Lysosome-associated membrane protein-2 (LAMP2)	3	179
SCRB2_HUMAN	Lysosome membrane protein 2	3	171
IL3RA_HUMAN	Interleukin-3 receptor subunit alpha (IL3RA)	3	159
CD86_HUMAN	CD86 (B7.2)	3	144

L-540 Cells

Protein Accession Number (Swissprot)	Protein Name	Peptides matched	Total Ion Score
S4A7_HUMAN	Sodium bicarbonate cotransporter 3	9	412
B3A2_HUMAN	Anion exchange protein 2	7	316
DPP4_HUMAN	Dipeptidyl peptidase 4	7	309
4F2_HUMAN	CD98 heavy chain	4	213
AAAT_HUMAN	Neutral amino acid transporter B(0)	4	187
CD45_HUMAN	Leukocyte common antigen CD45	4	172
LAMP1_HUMAN	Lysosome-associated membrane protein-1 (LAMP1)	3	203
LAMP2_HUMAN	Lysosome-associated membrane protein-2 (LAMP2)	3	169
